# *Shigella* and childhood stunting: Evidence, gaps, and future research directions

**DOI:** 10.1371/journal.pntd.0011475

**Published:** 2023-09-12

**Authors:** Karoun H. Bagamian, John D. Anderson IV, Gabriela Blohm, Suzanne Scheele

**Affiliations:** 1 Bagamian Scientific Consulting, LLC, Gainesville, Florida, United States of America; 2 Department of Environmental and Global Health, University of Florida, Gainesville, Florida, United States of America; 3 Health Affairs Institute, West Virginia University, Morgantown, West Virginia, United States of America; 4 Center for Vaccine Innovation and Access, Washington, District of Columbia, United States of America; Yale University School of Medicine, UNITED STATES

## Abstract

Early childhood growth deficits have been shown to have lifelong health and economic impacts, yet their connection to one of their underlying causes, diarrheal diseases, has remained difficult to characterize. Identifying the processes and mechanisms that underlie this link has remained a challenge due to the complexity of the relationship and limitations in access to more advanced laboratory methods. In recent years, however, several large-scale, multisite studies have extensively investigated and reported the prevalence, etiology, and impacts of diarrheal diseases in children under 5 years (CU5) in low- to middle-income countries (LMICs). These studies, in combination with several single-site studies, have applied more advanced laboratory methods to uncover the etiology, true prevalence, infection mechanisms, and inflammation biomarkers of diarrheal disease. Of the multiple pathogens that have been shown to be strongly associated with diarrheal disease in CU5, *Shigella* is one of the more prevalent and impactful of these pathogens. In this narrative review, we highlight key insights from these studies and identify knowledge gaps and directions for future research. According to these studies, *Shigella* is most commonly detected in toddlers and young children; however, it can cause more severe disease and has a greater impact on linear growth for infants. *Shigella* often has a stronger relationship to linear growth faltering (LGF) than other enteropathogens, with higher *Shigella* loads resulting in greater growth deficits. Future studies should employ more *Shigella*-specific molecular assays and identify diarrheal etiologies using standardized diagnostics to improve child anthropometric and *Shigella* surveillance. Also, they should focus on uncovering the mechanisms of the relationship underlying *Shigella* and growth faltering to better characterize the role of asymptomatic infections and intestinal inflammation in this relationship.

## Author summary

For decades, research has shown that growth deficits in early childhood can have long-term impacts on human health and economic potential. *Shigella* spp are a group of bacterial pathogens that commonly cause diarrhea in children under 5 years of age. Early studies suggest that infection with *Shigella* can reduce childhood growth, but the biological and developmental processes underlying this link remain uncharacterized. Since the early 2000s, there has been a global effort to investigate the prevalence, geographic distribution, and impacts of diarrheal infectious agents on early childhood growth. With the introduction of advanced molecular laboratory methods and the completion of several international collaborative studies, these efforts have yielded important insights. The studies have identified that, of these agents, *Shigella* spp often have a stronger relationship to early childhood growth than other pathogens. Our review distills the evidence for this relationship to identify key discoveries, knowledge gaps, and future research avenues. According to these studies, *Shigella* often has a stronger relationship to early childhood growth than other pathogens, and most children from high-risk populations have been infected with *Shigella* by age 5. The severity of the illness is greater in infants, though *Shigella* incidence is highest in toddlers and in children closer to 5 years old. Asymptomatic cases of *Shigella* can also give rise to stunting, highlighting the need to refine existing tools for clinical and laboratory surveillance. *Shigella* is also linked to intestinal inflammation indicators, warranting further research on how this biological pathway may influence growth. Addressing these research gaps may help better characterize the *Shigella*–child growth connection, thus enabling the potential reduction of early childhood growth deficits linked to *Shigella* infection.

## Introduction

Since the 1970s, evidence for the negative impacts of diarrheal disease on the growth of children under 5 years of age (CU5) has continued to accumulate. Although childhood diarrheal morbidity and mortality have declined substantially during the past 40 years [1], an estimated 149.2 million children globally still suffer from growth deficits that include linear growth faltering (LGF) and stunting (see Box 1 for definitions) [2]. Growth deficits in CU5 can indicate their risk for physical and cognitive developmental delays, morbidity, and mortality from infectious diseases early in life and from noncommunicable diseases later in life [3–5].

**Box 1. Definitions of important terms and concepts included in the review**

- **Linear growth faltering (LGF)** is when a child’s height/length falls below the expected growth curve, based on World Health Organization (WHO) Child Growth Standards. This term is most commonly used when linear growth deficits are evaluated using continuous child length or height-for-age Z score (LAZ; HAZ) outcomes. Note: recumbent length is measured in children under 2 years, and height is measured in children between 2 and 5 years old.
- **Stunting** is when a child’s LAZ or HAZ is more than 2 standard deviations below the WHO Child Growth Standards median. This term is used when LGF is described as a dichotomous categorical outcome.
- **Environmental enteric dysfunction (EED)** refers to subclinical inflammation of the intestinal tract and its components.

*Shigella*-attributable diarrhea was first linked to growth faltering almost 35 years ago in a seminal study by Black and colleagues on the role of infectious diseases in child nutrition [6]. Between the 1980s and early 2000s, many single-site studies investigated the etiology and prevalence of diarrheal infectious diseases, identifying more than 20 bacterial and viral pathogens that were linked to diarrhea. Most of these studies led to divergent and sometimes contradictory conclusions about which infectious diseases were most strongly linked to growth deficits, calling for the need to standardize research protocols and improve diarrheal disease and growth metrics. During the past 15 to 20 years, a series of large-scale studies have addressed these issues, clarifying our knowledge of the prevalence, etiology, and health impacts of diarrheal diseases on LGF and stunting in CU5.

Of the bacterial enteric pathogens, *Shigella* spp are most commonly associated with childhood growth deficits, as well as with proposed biomarkers of environmental enteric dysfunction (EED, see Box 1). In this review, we cover the most recent and relevant evidence for a relationship between *Shigella* infection and linear growth deficits in infants and toddlers and stunting in children aged 2 to 5 years old. We emphasize findings from large-scale, multisite studies, while including results from other single-site or smaller scale studies. We identify knowledge gaps regarding this relationship and propose future research directions.

## Methods

For our narrative review, we conducted a systematic search in 5 databases (Pubmed, Google Scholar, Web of Science, Embase, and Global Health CAB Direct). Per Bramer and colleagues, we limited our Google Scholar search to the first 200 most relevant records [7]. We used 2 broad search terms that included different combinations of relevant search strings.

- **Search term 1 (ST1) was geared to find studies regarding diarrhea and stunting or linear growth deficits. ST1:** Diarrhea and stunting: (“diarrhea” OR “diarrea” OR “diarrhoea”) AND (“stunting” OR “height for age” OR “height-for-age” OR “height” OR “Z score” OR “growth faltering” OR “linear growth” OR “malnutrition” OR “underweight”) AND (“child*”) AND (“outcomes” OR “changes”).
- **Search term 2 (ST2) was geared toward uncovering studies about symptomatic and asymptomatic *Shigella* in CU5. ST2:** “*Shigella*” or “shigellosis”) AND (“child*”) AND (“morbidity” OR “illness” OR “infection” OR “asymptomatic” OR “symptomatic” OR “stunting” OR “episodes” OR “events” OR “cases” OR “growth faltering” OR “linear growth” OR “malnutrition” OR “underweight” OR “height-for-age” OR “height for age”) AND (“cause” OR “etiology” OR “aetiology”).

**Fig 1:**
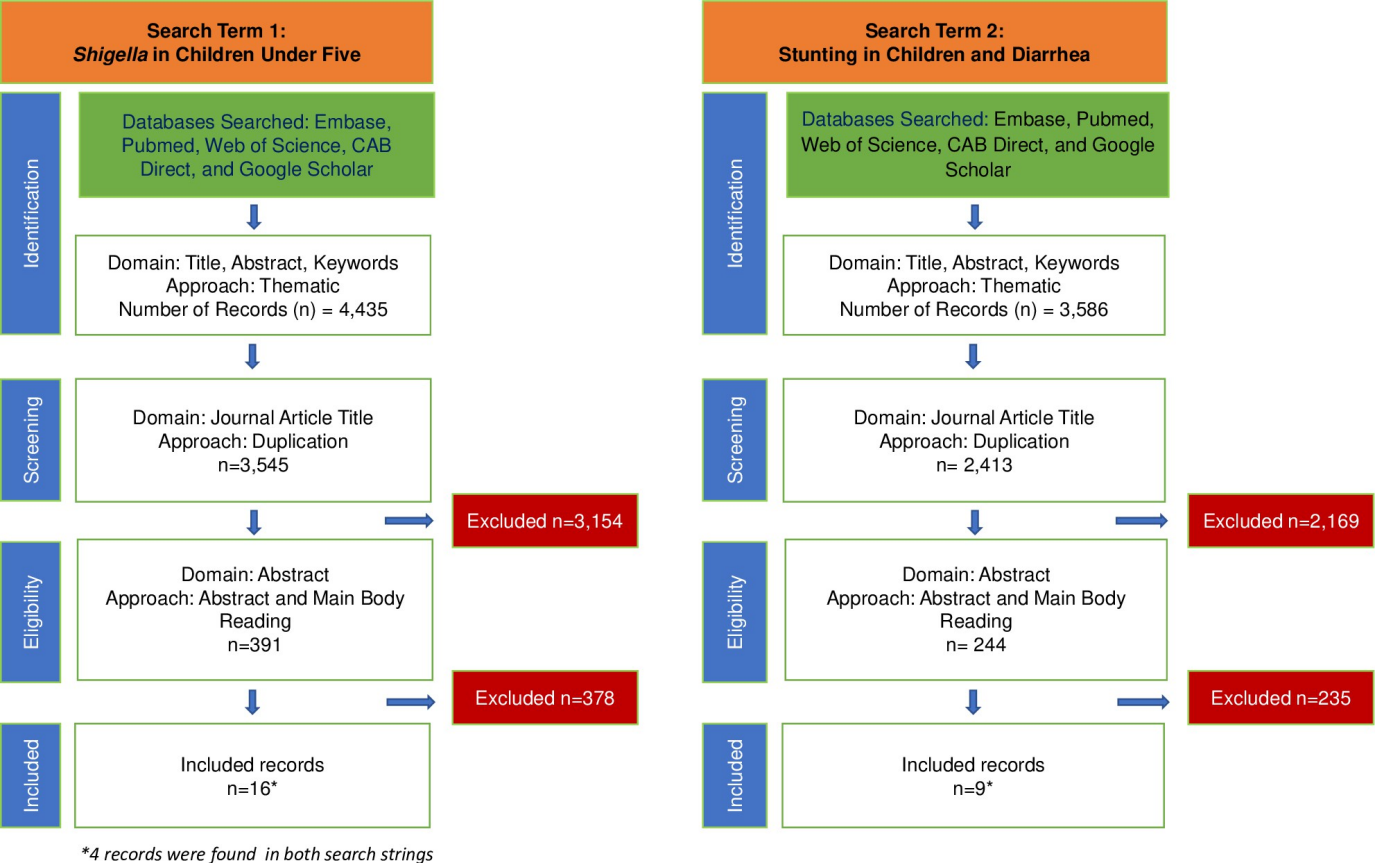
Flowchart of search process.

We identified 2,649 records for ST1 and retained 1,691 records after dropping duplicate entries. We identified 4,088 records for ST2 and retained 3,351 records after dropping any duplicate entries. We reviewed these studies for specific mentions of *Shigella* and whether the authors found a relationship with our outcomes of interest (stunting or linear growth deficits) (see Fig 1). In our scoping of these obtained publications, we also found mentions of other relevant studies and reviewed these publications (*n* = 46). We included any studies that explored diarrheal etiology in relation to stunting, linear growth deficits, or EED. We closely reviewed these studies for specific mentions of *Shigella* and whether the authors found a relationship with our outcomes of interest (stunting or linear growth deficits or EED markers). When no publications that related the presence of *Shigella* infection to a topic of interest to our study (e.g., data on less severe diarrhea [LSD] or depicting the importance of age at infection to LGF) were available, we instead included results from publications that related diarrhea to these outcomes of interest. This procedure resulted in the inclusion of 42 studies, which were used for context and synthesis.

### *Shigella* epidemiology in young children: Insights from 2 multisite studies

The Global Enteric Multicenter Study (GEMS) and Malnutrition and Enteric Disease Study (MAL-ED) are 2 of the largest and most comprehensive studies of the prevalence, etiology, and long-term impacts of childhood diarrhea in high-risk, low- to middle-income countries (LMICs). GEMS, a prospective, matched, case-control study that spanned 7 LMICs (Kenya, Mali, Mozambique, The Gambia, Bangladesh, India, and Pakistan), investigated the incidence and etiology of acute diarrhea in over 22,000 CU5. The study resulted in the largest dataset on this topic to date, combining traditional microbiological and more advanced molecular methods for identifying key predictors of early childhood growth deficits in LMIC populations. MAL-ED, a longitudinal birth cohort study in 8 LMICs (Peru, Brazil, South Africa, Tanzania, Pakistan, India, Nepal, and Bangladesh) investigated environmental drivers and gut function biomarkers of reduced growth in over 1,200 children under 2 years old (CU2). This study yielded important insights into the true prevalence, severity, and growth-specific impacts of asymptomatic or subclinical diarrheal infections. These and other single-center studies, including animal model research, are also exploring the mechanisms by which enteric pathogens can affect growth for CU5.

Since the earliest studies on diarrheal diseases, *Shigella* spp have been one of the most commonly detected bacterial agents in CU5. In GEMS and GEMS 1A, *Shigella* spp were shown to be strongly associated with moderate-to-severe diarrhea (MSD) [8–10] and also LSD [10] (see S1 Table). Also, *Shigella*, along with rotavirus, *Cryptosporidium* spp, and heat-stable enterotoxigenic *Escherichia coli* (ST-ETEC), were shown to have strong quantity-dependent associations with diarrhea [8]. Notably, *Shigella* and rotavirus were present in the largest proportion of cases where diarrheal disease could be attributed to a single or primary (most abundant) pathogenic agent (Figure 4 in Liu and colleagues [8]). Further, a molecular analysis of a subset of GEMS case and control samples from 4 study sites reported that *Shigella* infections characterized as “high level” (>14,000 copies of the *ipaH* gene detected in stool) were significantly associated with MSD [11]. In a GEMS-affiliated study of CU5 in Manhiça, Mozambique, a strong, significant association was found between MSD and infection with *Shigella*, when compared to 12 other enteric pathogens, including *Cryptosporidium* spp, *Giardia* spp, and rotavirus [12].

*Shigella* incidence is often highest in young children (24 to 59 months) and toddlers (12 to 24 months) [9,10,13], with most children being exposed by age 2 or 5 years, depending on the study (with prevalence ranging between 75% and 90% [13–15], see S1 Table) in certain LMICs. Quantitative PCR analysis of the *ipaH* gene (in a subset of 2,611 stool samples from the GEMS study) has indicated that the association between *Shigella* quantity and the incidence of MSD can become stronger as the child’s age increases, plateauing at 36 months [16]. The same trend was found in a single-site study of children aged 24 to 59 months who presented with MSD [13]. With an adjusted odds ratio of 191 (95% CI: 23.80, 24,565.84), MSD in this age group had almost 200 times more odds of being attributed to *Shigella* infection than to the other infectious agents in the study [12].

Recent studies have uncovered that, while younger children, specifically those between 6 months and a year, may not have the highest *Shigella* prevalence, they may experience a more severe illness and long-term sequelae from infection. For example, Rogawski and colleagues (2020) found that, while *Shigella* prevalence and quantity increased with age in children from the MAL-ED sites, it presented differently according to age and could be more severe for children in their first years of life [13]. In a recent post hoc analysis of etiology-agnostic MSD and child growth in CU2 from the original GEMS cohort, children who presented with any-cause MSD within their first year of life experienced more significant linear growth deficits at follow-up (60 days, range: 50 to 90 days) than children aged 12 to 23 months [17]. Specifically, when presenting with MSD, children aged 6 to 11 months experienced a significantly greater length-for-age Z score (LAZ) loss (0.07 LAZ) than children aged 12 to 23 months at follow-up, and children aged 0 to 6 months who experienced MSD exhibited more severe LGF (defined as a loss of 0.5 LAZ) than children 12 to 23 months at follow-up. While this study’s findings are not specific to *Shigella*-attributable diarrhea [17], taking these results together suggests that experiencing MSD as an infant is potentially more consequential than experiencing it as a toddler.

*Shigella* incidence also varies considerably by geographic location [8,13]. For example, Rogawski and colleagues (2020) found that *Shigella-*attributable diarrheal incidence varied substantially across study sites, ranging from as low as 5.4 cases per 100 child-years (95% CI: 2.9, 10.0) in South Africa to 75.1 cases per 100 child-years (95% CI: 66.7, 84.5) in Bangladesh [13]. For a summary of the main conclusions about the age-specific prevalence and severity of *Shigella*, see Box 2.

**Box 2. Key conclusions**

- **Most MSD cases in toddlers and children are attributable to *Shigella* spp.**
- ***Shigella* spp–attributable diarrhea is more common in toddlers (aged 12 to 23 months) and young children (aged 24 to 59 months) than in infants (aged 0 to 11 months).**
- **While *Shigella* infection is less common in infants (aged 0 to 11 months), it can result in severe illness and may be more consequential for future LGF in infants than for toddlers and children aged 24 to 59 months).**
- ***Shigella* spp incidence can vary significantly according to age and geographic location.**

### Symptomatic *Shigella* infections and decreased linear growth

Bacteria- or protozoa-attributable diarrhea or enteric infections have been associated with greater subsequent linear growth deficits than enterovirus infections or attributable diarrhea [18,19]. The bacterial pathogens most often reported to have a connection to stunting are *Shigella* spp, *E*. *coli pathovars* (*E*. *coli* spp), and *Campylobacter* spp [18,20–22]. Other pathogens that also show relationships to LGF or stunting include *Cryptosporidium*, *Giardia*, norovirus, and *Enterocytozoon bieneusi* (*E*. *bieneusi*), a microsporidian [18,22,23]. For example, Rogawski and colleagues (2018) found that while *Shigella* spp and *E*. *bieneusi* were associated with the largest LAZ decrease per log increase in gram of stool, subclinical infections of enteroaggregative *Escherichia coli* (EAEC), *Campylobacter* spp, and *Giardia* spp were also negatively associated with linear growth [18].

Black and colleagues (1984) was the first study to pinpoint that *Shigella*-attributable diarrhea was associated with decreased childhood linear growth [17]. Two decades later, Lee and colleagues (2014) reported the same association in a population of 0- to 6-year-old children from the Peruvian Amazon [24]. Chiefly, *Shigella*-attributable diarrhea was significantly associated with decreased linear growth, indicating these findings’ replicability in a geographically distinct population (Table 1).

In a recent pathogen-specific analysis of the original GEMS MSD samples, untreated *Shigella* spp, *Cryptosporidium* spp, typical enteropathogenic *E*. *coli* (EPEC), and ST-ETEC were associated with linear growth declines during the first 2 years of life [22]. *Shigella*-positive toddlers (aged 12 to 24 months) who were treated with antibiotics exhibited a 4-fold decrease in LGF as compared to untreated *Shigella-*positive toddlers, indicating that treatment with WHO-recommended antibiotics (primarily ciprofloxacin) can ameliorate stunting impacts of *Shigella* infection. While the above studies used microbiological methods to assess diarrheal etiology, most studies now rely on more sensitive molecular detection methods (chiefly quantitative PCR). Further characterizations have been made to describe associations between *Shigella*-attributable diarrhea and LGF [18,19]. For example, Rogawski and colleagues (2018) found that *Shigella*-attributable diarrheal episodes were associated with LAZ decreases in children (−0.03; 95% CI: −0.05, 0.00) as early as 3 months [18]. When considering pathogen quantity, *Shigella* had the greatest association with LAZ decreases. In the children who had follow-up anthropometric data at age 5 years (65% of the study population), only *Shigella-* or norovirus-associated growth decrements were sustained or increased from their last measurement at age 2. For all other pathogens that showed a significant relationship between their burden and decreased linear growth at age 2, this association diminished at age 5 [18]. As for single-site studies, a longitudinal birth cohort study of CU2 in Bangladesh recently reported that experiencing *Shigella*-attributable diarrhea was associated with a −0.12 LAZ (95% CI: −0.26, 0.03) decrease at 12 months of age [19], similar to results found in Rogawski and colleagues (2018) [18].

**Table 1:** Studies exploring a relationship between *Shigella* infection and decreased linear growth/stunting or growth measures in young children and other relevant outcomes.

Author, year	Study site(s), period	Study Design	Study population	Methods	Growth faltering—related measure	Main outcomes pertinent to review	Limitations
**Nasrin and colleagues, 2022 [25]**	MAL-ED sites.^a^ 2009−2012	Multi-country birth cohort study	1,715 CU2	• Non-diarrheal stools collected monthly and assessed using TaqMan Array Cards (Thermo Fisher, Waltham, Massachusetts, United States of America) • Used GEEs to assess associations between asymptomatic infection and nutritional status while adjusting for covariates (e.g., sex, maternal height, site, and an SES index composed of water/sanitation, assets, maternal education, and income indicators)	WAZ LAZ CIAF	Asymptomatic infection was significantly associated with stunting (aOR 1.60; 95% CI: 1.50, 1.70), wasting (aOR 1.26; 95% CI: 1.09, 1.46), underweight (aOR 1.45; 95% CI: 1.35, 1.56), and CIAF (aOR 1.55; 95% CI: 1.46, 1.65) in all the study sites, except Brazil	• Not a *Shigella*-specific molecular assay; allows cross-detection of EIEC • Other residual confounders not captured by covariates examined in the study could lead to non-causal associations
**Nasrin and colleagues 2021 [22]**	GEMS sites,^b^ 2007−2011	Case	220 children with clinically severe MSD (aged 0 to 59 mo) per year, per each of 7 sites: 8,077 total cases in 7,545 children	• Collected clinical and epidemiological data, anthropometric measurements, and fecal samples at enrollment and 60-day follow-up • Stool specimen analyzed by microbial methods [26]; focused on MSD pathogens^d^ HAZ changes calculated using linear mixed-effects regression model	LAZ for CU2 HAZ for children 2 to 5 years old	• Untreated *Shigella* was one of 3 pathogens significantly associated with linear growth decline for infants aged 0 to 11 months • Children aged 12 to 23 months with *Shigella*-positive dysentery treated with WHO-recommended antibiotics had improved linear growth • Similar, but not statistically significant, trend among infants with *Shigella*-positive dysentery and toddlers with *Shigella*-positive watery diarrhea	• Unmeasured events between enrollment and follow-up may have contributed to limited growth • Detrimental effects of MSD can be potentially overcome by catch-up growth • Observational study • Unable to measure antibiotic use compliance in treated CU2 *Shigella* cases
**Das and colleagues 2021[27]**	GEMS sites,^b^ 2007−2011	Case	1,394 CU5 from a total of 3,859 children enrolled at the Bangladesh site	• Collected clinical and epidemiological data, anthropometric measurements, and fecal samples at enrollment and 60-day follow-up • Stool specimen analyzed by microbial methods [26]; focused on MSD pathogens^d^ • Used GEEs to assess associations between *Shigella* and continuous anthropometric measures with exchangeable correlation and identity link functions	LAZ for CU2 HAZ for children 2 to 5 years old WAZ WHZ	• Negative association between *Shigella* and WHZ (aOR −0.11; 95% CI −0.21, −0.001); relationship remained after adjustment for covariates • No significant relationship between *Shigella* and HAZ or WAZ • Higher proportion of children with MSD and *Shigella* infection were stunted, wasted, or underweight	• No differentiation of species-level effects of *Shigella* and *Campylobacter* infections • Results from 1 subdistrict in Bangladesh may not be nationally generalizable • Unable to determine the relationship between maternal BMI and age, gestational age, and birth weight and child growth failure
**Rogawski and colleagues 2018 [18]**	MAL-ED sites,^a^ 2009−2012	Secondary stool analysis from longitudinal community birth cohort	35,622 stool samples from 1,469 CU2	Anthropometric measurements, monthly stool and diarrheal stool samples collected until 2 y; one follow-up visit at 5 y GEMS TaqMan (Liu and colleagues, 2016 [8]) for enteropathogen surveillance	LAZ	• Subclinical infection by *Shigella* and other pathogens and their quantities were more consequential for linear growth than diarrheal episodes • Bacteria and parasites were associated with small decreases in child length after 3 mo and at 2 y • LAZ reduction in subclinical infection with *Shigella* (−0.14); other pathogen effects ranged from −0.1 (*Giardia*) to −0.21 (EAEC) for those with a substantial effect • *Shigella* and *E*. *bieneusi* were associated with the largest decreases in LAZ per 1 g of stool: *Shigella* (−0.13 LAZ, 95% CI:−0.22,−0.03); *E*. *bieneusi* (−0.14 LAZ, 95% CI:−0.26, −0.02)	• Not a *Shigella*-specific molecular assay; allows cross-detection of EIEC • Longitudinal model estimates were less precise compared to the height attainment model • A small number of children were sampled at each site • There could be infections that were severe at the individual level that have limited impact at the population level • Potential underestimate of the growth impact given diarrheal cases were mild
**Platts-Mills and colleagues 2017 [28]**	MAL-ED site (Mirpur, Bangladesh), 2009−2012	Matched community case-control	• Cases: 486 malnourished children (WAZ < −2) aged 6 to 23 mo • Controls: 442 normal-weight children (WAZ > −1) aged 6 to 23 mo	• Stools collected at enrollment and, after a 5-mo nutritional intervention for cases • Custom qPCR TaqMan Array Card for 32 enteropathogens	WAZ	• *Shigella* associated with malnourished cases • Second highest odds ratio of the 5 other pathogens associated with malnourishment (*Shigella*/EIEC OR: 1.65; 95% CI: 1.10, 2.46; *Giardia* OR: 1.73; 95% CI: 1.20, 2.49) • Total burden of these pathogens remained associated with malnutrition after adjusting for SES factors	• Broad definition of malnourished children may limit specificity of associations • Case-control design limits the ability to illustrate temporal associations between enteropathogen infection and malnutrition • Unable to determine if malnutrition is a cause or sequelae of enteropathogen carriage, especially in immune-suppressed children • Findings may not be generalizable to other LMICs
**Bona and colleagues 2019 [29]**	Six cities^c^ from Northeastern Brazil, 2009−2012	Community cross-sectional case-control	1,200 children aged 2 to 36 mo from low-SES communities (presenting in healthcare units or during active surveillance)	• Collected SES, nutritional status, and clinical information • Stool samples tested for *Shigella*/EIEC using PCR • Tested positive samples for 28 VRGs with multiplex PCR	HAZ	• Detection of *Shigella* virulence gene *virB* was associated with low HAZ, regardless of whether a child had diarrhea • The mean HAZ score for children in which the *virB* gene was detected was −1.26 less than in children in which the gene was not detected	• Small sample size relative to other studies: 60 children were evaluated; 42 were cases • Because of the molecular assays used (Luminex bead-based assays/conventional multiplex PCR), sensitivity was an order of magnitude lower than qPCR
**George and colleagues 2018 [14]**	ICDDR, B GEMS DSS (Mirzapur Upazila, Bangladesh), 2014	Prospective community cohort	203 children, 6 to 30 mo	• Stool samples and anthropometric measurements collected at baseline and 9 mo follow-up • GEMS TaqMan (Liu and colleagues, 2016 [8]) for enteropathogen surveillance • ELISA assays to measure environmental enteropathy markers	HAZ	Children with *Shigella* had 2× higher odds of being stunted at baseline and follow-up	• Not a *Shigella*-specific molecular assay; allows cross-detection of EIEC • Did not assess parasites in soil • Did not collect blood samples or measure enteropathogen antibodies
**Schnee and colleagues 2018 [19]**	PROVIDE (Mirpur, Dhaka, Bangladesh), 2011−2013	Longitudinal birth community cohort	521 CU2, 1,742 episodes of diarrhea	• Anthropometric measures collected at enrollment and predetermined time points (12, 24, 40, 52, and 104 weeks of age) • Biweekly assessments of diarrheal episodes (stools were collected) and other factors until 2 y • Serum collected for EED marker testing • GEMS TaqMan (Liu and colleagues, 2016 [8]) for enteropathogen surveillance	LAZ at 12 mo	• Found that linear growth and diarrhea relationship was etiology-dependent • *Shigella*/EIEC: third highest LAZ change per attributable episode:−0.12; 95% CI:−.26, 0.03 • No relationship between number of episodes or diarrhea duration with LAZ at 12 mo	• Not a *Shigella*-specific molecular assay; allows cross-detection of EIEC • Findings may not be generalizable to other LMICs • Non-diarrheal stool not collected, potential underestimate of growth effects by asymptomatic infections • Stool collection biased towards long-duration diarrhea
**Vonaesch and colleagues 2018 [21]**	AFRIBIOTA (Bangui, Central African Republic and Antananarivo, Madagascar), 2016	Cross-sectional	• 404 fecal samples from children (aged 2 to 5 years) recruited for AFRIBIOTA project • 46 duodenal and 57 gastric samples from CU5 with moderate and severe stunting	• 16S ribosomal RNA sequencing/semiquantitative culture methods • Culture of duodenal aspirates to detect SIBO	HAZ at enrollment	• *Shigella flexneri*/*E*. *coli* overrepresented in stool samples from children exhibiting stunted growth—7.6-fold change between cases and control (only other overrepresented pathogen was *Campylobacter* spp) • Overrepresentation of *Shigella*/*E*. *coli* in children with moderate and severe stunting from CAR	• Cross-sectional design limits ability to determine causality • Did not collect gastric or duodenal samples from non-stunted controls; unable to compare these samples between children with stunting and normal growth • Low abundance taxa might have been missed • No confirmation that oral species were also in the CU5s’ mouths • Majority of samples from children from CAR (62%)
**Lee and colleagues 2014 [24]**	Peruvian Amazon, 2002−2006	Prospective, longitudinal community	443 CU6; 3,711 stool samples	• Diarrheal surveillance 3 times/week and monthly anthropometric surveillance (extension of Black and colleagues, [6]) • Bacterial culture for enteropathogen surveillance; PCR testing of dysenteric stools for *ipaH* gene [30] • Mixed-effect models	Change in length over 9 mo	• *Shigella* diarrhea was associated with decreased linear growth (0.081 less growth per episode or 0.055 less linear growth per % days with *Shigella*) • Shorter episode duration than in Black and colleagues [6]	• Use of bacterial culture for surveillance may have resulted in underdetection of *Shigella* and other etiological agents • Effects of *Shigella* may have been less severe than other pathogens because more cases received effective antibiotic treatment
**Black and colleagues, 1984 [6]**	Bangladesh, 1978−1979	Longitudinal community surveillance	157 children 2 to 28 months	• Infectious disease surveillance every other day and monthly anthropometric surveillance (used local growth pattern reference curves) • Bacterial culture for enteropathogen surveillance • ANOVA and linear and stepwise multiple regression	Change in length status from start to end of study (1 y)	• Shigellosis had the strongest negative effect on bimonthly and annual linear growth • Shigellosis-related diarrheal episodes had a longer duration than other episodes associated with other measured enteropathogens • *Shigella* and ETEC were the most commonly detected enteopathogens (15% and 30%, respectively)	• Difficult to compare results to other populations because of location-specific growth metric (change in percentage of the village reference for age from start to end of study) • Use of bacterial culture for surveillance may have resulted in underdetection of *Shigella* and other etiological agents

^a^MAL-ED sites: Dhaka, Bangladesh; Fortaleza, Brazil; Vellore, India; Bhaktapur, Nepal; Loreto, Peru; Naushahro Feroze, Pakistan; Venda, South Africa; and Haydom, Tanzania.

^***b***^GEMS sites: Bamako, Mali; Manhiça, Mozambique; Nyanza Province, Kenya; Basse Santa Su, The Gambia; Mirzapur, Bangladesh; Kolkata, India; and Bin Qasim Town, Karachi, Pakistan.

^*c*^Cajazeiras (Paraiba), Crato (Ceará), Ouricuri (Pernambuco), Patos (Paraiba), Picos (Piauí), and Sousa (Paraiba).

^d^*Rotavirus*, *Cryptosporidium*, *Shigella* spp, typical EPEC, heat-stable ETEC, non-typhoidal *Salmonella* spp.

ANOVA, analysis of variance; aOR, adjusted odds ratio; BMI, body mass index; CAR, Central African Republic; CIAF, composite index of anthropometric failure; CU2, children under age 2 years; CU5, children under age 5 years; EAEC, enteroaggregative *Escherichia coli*; EIEC, enteroinvasive *E*. *coli*; EPEC, enteropathogenic *E*. *coli*; ETEC, enterotoxigenic *E*. *coli*; GEEs, generalized estimating equations; HAZ, length/height-for-age Z score; HFA, height-for-age Z score; LAZ, length-for-age Z score; mo, month(s); LMIC, low- to middle-income country; MSD, moderate-to-severe diarrhea; PCR, polymerase chain reaction; PROVIDE, Performance of *Rotavirus* and Oral Polio Vaccines in Developing Countries; qPCR, quantitative PCR; SES, socioeconomic status; SIBO, small intestinal bacterial overgrowth; WAZ, weight-for-age Z score; WHO, World Health Organization; WHZ, weight-for-height Z score.

### The importance of asymptomatic *Shigella* infections

Molecular diagnostic tools have led to the discovery that subclinical enteric infections are more important than previously realized. Across all MAL-ED study sites, subclinical infections had stronger negative and more significant associations with LAZ at 2 years than observed clinical diarrheal infections did [18]. Subclinical *Shigella* infections were associated with substantial decreases in child length at 2 years (LAZ: −0.14; 95% CI: −0.27, −0.01). In an analysis of asymptomatic infections from the MAL-ED study, asymptomatic *Shigella* infection had a significant association with stunting (adjusted odds ratio [aOR]: 1.90; 95% CI: 1.50, 1.70) after adjusting for relevant covariates (Table 1) [25].

In a molecular analysis of stool samples of the GEMS Kenya cohort, MSD-associated pathogens (*Shigella* spp, ST-ETEC, typical EPEC, non-typhoidal *Salmonella* spp, rotavirus, and *Cryptosporidium* spp) were detected in 20% of children not exhibiting diarrheal symptoms at enrollment (controls). Controls who had MSD-associated pathogens in their stool had significantly higher odds of stunting at their 60-day follow-up (OR 1.6, 95% CI: 1.1, 2.2), regardless of whether they developed diarrhea, as compared with controls without MSD-associated pathogens in their stools [31]. However, as is evident in this study, infections that appear to be asymptomatic (in cross-sectional studies or short-term sampling scenarios) may actually result in diarrhea at a later date: 39% of controls did develop diarrhea 14 days after enrollment. Although it remains difficult to ascertain whether an enteric infection in the field is truly asymptomatic or subclinical, recent experimental human challenge models for enterotoxigenic *E*. *coli* (ETEC) showed that experimental ETEC infections elicited inflammatory responses associated with stunting in asymptomatic and diarrheal cases [32]. Although this has not been shown experimentally for *Shigella*, these pathogens share similar characteristics when it comes to child health impacts, and this study provides preliminary experimental evidence of the impact of asymptomatic enteric bacterial infections on inflammatory pathways related to child growth.

Molecular diagnostics have also uncovered a high prevalence of *Shigella* infections in CU2. In the study cohort examined by Rogawski and colleagues (2018), 82% (*n* = 1,715) of children had been infected with *Shigella* by age 2, and prevalence was already quite high at multiple sites in the first year of life [18]. By 12 months of age, the cumulative incidence of *Shigella* infection in children was nearly 75% in Tanzania and approximately 40% in India and Bangladesh. A prospective cohort study of 6- to 30-month-old children in Bangladesh, noted that the study population had a high prevalence (76%) of asymptomatic enteric infections [14]. The authors detected *Shigella* in 35% of the studied children at baseline. Also, children with *Shigella* had significantly higher odds (OR: 2.2, 95% CI: 1.12, 4.38) of LGF at baseline and at their follow-up 9 months later (OR: 2.01, 95% CI: 1.02, 3.93). These high levels of infection indicate the potential ubiquity of *Shigella* within specific child populations, who often also display a higher propensity for growth faltering and coinfections by other enteric pathogens detrimental to child health and development.

In a study of the gastrointestinal microbiomes of children aged 2 to 5 years from the Central African Republic (CAR) and Madagascar, a higher enteropathogen prevalence was found in fecal samples from children with moderate to severe stunting than in control fecal samples [21]. This study population’s inclusion criteria required that children not be experiencing severe diarrhea (deemed as asymptomatic carriage by the authors). Of the enteropathogens detected, *Shigella flexneri*/*E*. *coli* were among the pathogen groups overrepresented in stool samples from children exhibiting stunting. In this study, pathogens were identified by 16S ribosomal RNA sequencing, which cannot differentiate between *S*. *flexneri* and *E*. *coli* species. Upon closer examination, only children from the CAR had an overrepresentation of *Shigella*/*E*. *coli*, potentially another example of high *Shigella* burden being location-specific. The fold change was twice as high for *Shigella*/*E*. *coli* than for *Campylobacter* spp (7.6-fold change versus 3.6-fold change between cases and controls), suggesting that *Shigella* and *E*. *coli* species have a stronger relationship to growth faltering than other pathogens, a finding common to other studies (Table 1). It is unclear how much of the observed difference between cases and controls is specifically from *Shigella* or *E*. *coli*, of which there are pathogenic and nonpathogenic forms. Most other studies using molecular methods have also used cross-reactive assays (see Tables 1, 2, and S1; discussed in *Cross-detection by molecular assays* section). Some researchers have determined ways to ascertain the proportion of different *Shigella* species included in their samples (see details in Platts-Mills and Rogawski McQuade [33]).

**Fig 2:**
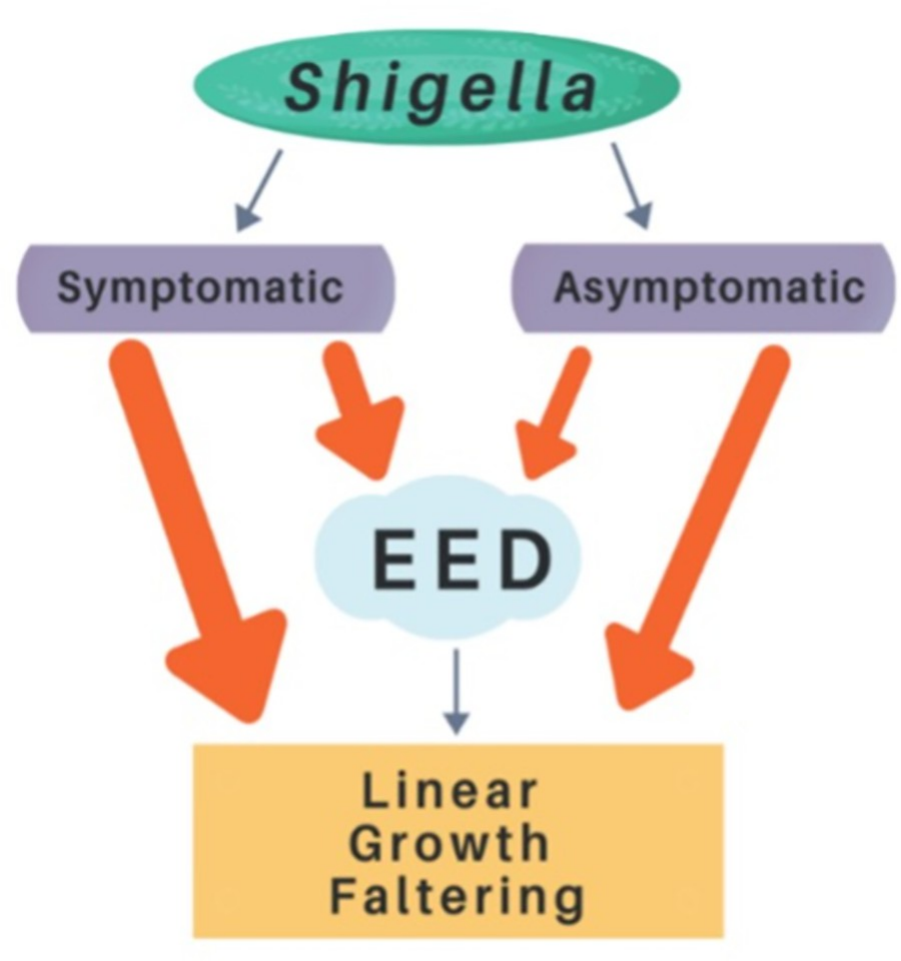
Synthetic diagram of evidence for the pathway between *Shigella* infection and childhood LGF and stunting. Red-orange arrows depict the associations relevant to this report. Each arrow’s thickness denotes how many published studies show support for that pathway. Diagram credit: C. Forsgren. EED, environmental enteric dysfunction; LGF, linear growth faltering.

As summarized in Fig 2, many studies have shown relationships between symptomatic and asymptomatic *Shigella* infections and LGF or EED, the primary proposed pathway of how enteric infections may result in LGF. Further research into the epidemiological importance of asymptomatic infections will improve existing knowledge of the connection between *Shigella*, EED, and LGF, shedding additional light on the relative importance of symptomatic and asymptomatic infections.

### Diarrheal severity and pathogen quantity in relation to diminished growth

In GEMS 1A, children with MSD and LSD both experienced significant LGF at their follow-up visit. However, when comparing the height-for-age Z score (HAZ) change for cases (patients with diarrhea) with their matched controls, while children with MSD in the older age classes (12 to 23 months and 24 to 59 months) experienced a somewhat greater decrease in HAZ as compared to those who experienced LSD, these differences were not statistically significant. Children with MSD had a significantly greater HAZ reduction than children with LSD only for the youngest age class (0 to 11 months) [10]. The results focused on the relationships of all etiology LSD and MSD to stunting, which include *Shigella* spp, among other pathogens. The findings about MSD and LSD, coupled with the results about asymptomatic infections also impacting linear growth, suggest that enteric infections need not be severe to leave their mark on a child’s physical development.

In studies by MAL-ED investigators (2017) and Rogawski and colleagues (2018), asymptomatic enterobacterial infections (including *Shigella*) were associated with greater LAZ decreases than diarrhea-associated enterobacterial infections [18]. However, in Rogawski and colleagues (2020), increases in the quantity of *Shigella* detected in a patient’s sample were associated with greater declines in LAZ [13]. *Shigella* was one of 2 pathogens with the largest LAZ decrease (LAZ, −0.13; 95% CI: −0.22, −0.03) per log increase in gram of stool (the other being *E*. *bieneusi*) [18]. Lindsay and colleagues (2015) showed that approximately 70% of case and control (no diarrheal symptoms when sampled) children with high *Shigella* levels in their stool also displayed moderate-to-severe stunting (HAZ ≥ –2 SD) [16] (Table 1). As higher pathogen loads have an apparent relationship to linear growth even when not accompanied by symptoms, these findings suggest that pathogen quantity is also an important factor to consider concerning childhood growth.

### Studies that did not report a relationship between *Shigella* and LGF

Our search uncovered 5 studies that reported either nonsignificant or negative associations between *Shigella* infection and LGF-related factors [15,27,28,31,34]. Although most of these studies were not designed to directly investigate the link between *Shigella* and LGF, they are worth noting, as they point to other factors that might explain observed differences in the relationship between enteric infections and LGF. In the Madagascar-based AFRIBIOTA study that characterized the etiology of small intestinal bacterial overgrowth (SIBO) and its relationship to stunting in children 2 to 5 years of age, the prevalence of *Shigella* was not significantly associated with LGF [15]. In this study, *Shigella* spp were present in 83.3% of the population. The authors mentioned that reinfections and postinfection shedding were of unknown occurrence, and in the absence of longitudinal data, the lack of association between *Shigella* infection and stunting should be considered with caution. In a post hoc analysis of GEMS samples from Bangladesh, *Shigella* spp were negatively associated with weight-for-height Z scores, but not with HAZ scores [27]. Donowitz and colleagues (2021) conducted a secondary analysis of child stool samples from a longitudinal birth cohort study in Dhaka and found that socioeconomic indicators had a greater impact on growth and neurodevelopment than most pathogens studied (including *Shigella* spp) [34]. A cross-sectional secondary analysis of stool samples from an open cohort, cluster-randomized controlled trial in Dhaka, Bangladesh, revealed that, of the 15 pathogens studied (including *Shigella* spp), only *Giardia* spp were associated with lower HAZ scores and stunting [23]. These 4 studies were conducted in Bangladesh—which is a MAL-ED study site and is also the location of 2 other single-site studies [14,19] that did find associations between *Shigella* and stunting and LAZ decrements (Table 1). These studies all suggest that the geographic scope of a study, sampling design, socioeconomic factors, and coinfection with other pathogens could affect a study’s conclusions about the strength of the relationship between *Shigella* and childhood growth. Key conclusions about the relationship between *Shigella* infection and childhood growth are summarized in Box 3.

**Box 3. Key conclusions**

- ***Shigella* infection can result in LGF in young children.**
- ***Shigella* often has a stronger relationship to LGF than other enteropathogens.**
- **Asymptomatic *Shigella* infections also result in linear growth decrements.**
- **By age 2, most young children in certain LMICs have experienced *Shigella* infections.**
- **MSD and LSD are associated with LGF for toddlers and children, and this relationship does not vary significantly by diarrheal severity.**
- **MSD is associated with a greater decrease in linear growth than LSD for infants, but not for toddlers and children.**
- **Higher *Shigella* loads result in greater growth deficits.**

### *Shigella* and EED

*Shigella* and other commonly detected pathogenic enteroinvasive bacteria, such as *Campylobacter* spp and EAEC, can be particularly disruptive to the intestinal tract [35]. Current consensus is that enteric infections may lead to EED, a condition characterized by subclinical inflammation of the intestinal tract and atrophy of its components. Most often identified in young children from settings with low access to improved water and sanitation and adequate nutrition, EED is linked to undernutrition in CU5 and is thought to be a distal cause of linear growth deficits and childhood stunting.

While EED is the proposed pathway for how enteric infections lead to childhood linear growth decrements, it does not have a universal case definition, there is no current consensus on accepted diagnostic tests or criteria, and multiple hypotheses exist about how it leads to childhood stunting or linear growth decrements [36]. In general, EED is characterized in young children as a subclinical malfunctioning of the gastrointestinal system resulting from consuming resources with high fecal pathogen levels, which can exacerbate undernutrition. However, differing opinions and evidence exist about how this occurs. Five potentially associated mechanisms have been posited to explain how EED emerges: (1) increased permeability of the intestinal tract that results in chronic systemic inflammation from translocation of bacteria or pathogen fragments into the child’s systemic circulation; (2) chronic inflammation not related to pathogen translocation; (3) decreased absorption; (4) disruption of the endocrine system; and (5) dysbiosis (or microbiome disruption) [36].

The traditional EED test involves measuring certain sugar levels and calculating their ratios (lactulose: mannitol ratio or lactulose: rhamnose ratio) as proxy measures of gut function (permeability). While these ratios have been shown to have a relationship to linear growth decrements in young children, this assay is cumbersome, and its results can be inconsistent. Therefore, and because of the varying hypotheses and case definitions of EED, numerous proposed EED markers exist (approximately 50 [36]). Most current etiology-specific EED studies often investigate a suite of EED- and inflammation-related biomarkers. In general, they have found associations between their studied pathogens and a few of these markers of interest. While detecting these associations is infrequent, *Shigella* is one of few surveyed enteric pathogens associated with EED markers in multiple studies.

*Shigella* detection was associated with the gut inflammatory marker myeloperoxidase (MPO) in 3 recent studies [13,37,38]. MPO is an enzyme primarily expressed in neutrophils, which can result in localized tissue damage and inflammation upon activation. Rogawski and colleagues (2020) found a linear dose-response relationship between *Shigella* quantity (as determined by qPCR) and MPO concentrations (Table 2) [13]. Stools containing *Shigella* had higher MPO levels, and the association between MPO and *Shigella* quantity was greater in diarrheal than in non-diarrheal stools. *Shigella* infection was also associated with the systemic inflammation marker α-1-acid glycoprotein (AGP), with its levels being elevated in the presence of *Shigella* infection in a dose-dependent manner. Bona and colleagues (2019) studied *Shigella*-associated virulence-related genes (VRGs) in Brazilian children, finding that MPO levels were consistently higher in child stool samples positive for 6 VRGs (*ipgB1*, *ipgB2*, *ospF*, *sen/ospD3*, *virA*, and *virB*) when compared with children negative for each of these VRGs [29]. *Shigella* infections were strongly associated with increased MPO in a secondary analysis of stools from CU5 with clinical symptoms in Zambia [37]. Also, this study reported strong associations between *Shigella* spp detections and an overall measure of environmental enteropathy (EE) score (see Table 2), further supporting its role in EED.

The connection between *Shigella* and MPO is especially intriguing, considering that MPO is an EED biomarker previously shown to be associated with decreased linear growth and weight-for-age (WFA) and body mass index (BMI). In 2 EED and childhood growth studies from MAL-ED sites, MPO (one of the 3 biomarkers studied) had more consistent, robust relationships to observed growth deficits [39,40]. In Bangladesh, children with high MPO levels during their second year of life experienced significant linear growth losses [39]. In Peru, MPO maintained its significant relationship to LAZ throughout model refinements and explained more of the observed LAZ variances than the other 2 surveyed biomarkers [40]. Richard and colleagues (2019) investigated the relationship between EED markers and growth in a subset of the MAL-ED cohort with complete relevant data at age 5 (*n* = 1,107). They found that increased MPO levels were associated with decreased WFA and BMI Z scores at age 5 [41]. The relationship of *Shigella* to a biomarker that has been repeatedly linked to growth decrements in children implies a potential connection between *Shigella*, EED, and childhood stunting. However, other studies have shown no relationship of MPO to stunting. George and colleagues (2018) did not find a connection between *Shigella* (or other pathogenic bacteria except ETEC) with EED markers or EE scores in their cohort of young children (children aged 6 to 30 months) (Table 2) [14].

Other studies have shown relationships between *Shigella*, other inflammation-related markers, and linear growth deficits. Schnee and colleagues (2018) found that *Shigella*-attributable MSD was significantly associated with increased serum C-reactive protein (CRP), which is a measure of systemic inflammation. *Campylobacter* spp were the only other enteric pathogens showing a similar relationship. This study also reported a trending association between increased systemic inflammation and LGF (see Table 2) [19].

**Table 2:** Studies showing a relationship between *Shigella* infection and EED.

Author, year	Study site(s), period	Study design	Study population	Methods	EED-related measure	Main outcomes	EED result–specific limitations
**Gazi and colleagues, 2022 [38]**	Bangladesh Environmental Enteric Dysfunction (BEED) study (Mirapur, Bangladesh), 2017	Community-based intervention cohort study	1,050 stunted and at-risk of stunting children between 12 and 18 mo of age	• Estimated the efficacy of a nutrition intervention to investigate the significance of enteric pathogens in the EED pathophysiology • Formed and validated histological EED scoring • TaqMan Array Cards (Thermo Fisher, Waltham, Massachusetts, USA) based on GEMS TaqMan (Liu and colleagues, 2016 [8]) for enteropathogen surveillance	MPO, AAT, NEO, CALP, and Reg1B	• MPO and CALP levels significantly higher in *Shigella*/EIEC-positive participants (*P* = 0.05 and *P* = 0.02) • No significant relationship between *Shigella*/EIEC detection and HAZ, WAZ, and WHZ	• Not a *Shigella*-specific molecular assay; allows cross-detection of EIEC • All of the children in the study were malnourished; limits some interpretations without non-malnourished controls • Could not adjust for other factors (e.g., breast feeding status, birth weight, pathogen burden, and maternal height) that have been shown to be important in other studies
**Rogawski McQuade and colleagues, 2020 [13]**	MAL-ED sites,^a^ 2009–2012	Secondary stool analysis from longitudinal community birth cohort	41,450 stool samples from 1,715 CU2	• Monthly stool and diarrheal stool samples • GEMS TaqMan (Liu and colleagues, 2016 [8]) for enteropathogen surveillance	MPO, NEO, AAT, ACP	• *Shigella* had a dose-dependent response to MPO (0.33 log [ng/mL]; 95% CI: 0.27, 0.40). • Stools with *Shigella* had slightly elevated ACP levels	• Not a *Shigella*-specific molecular assay; allows cross-detection of EIEC
**George and colleagues, 2018 [14]**	ICDDR, B GEMS DSS (Mirzapur Upazila, Bangladesh), 2014	Prospective community and household cohort	203 children aged 6–30 mo	• EE score from ELISA • GEMS TaqMan (Liu and colleagues, 2016 [8]) for enteropathogen surveillance	AAT, MPO, NEO, CALP	EE fecal markers only had a relationship to ETEC, not *Shigella* or others	• Not a *Shigella*-specific molecular assay; allows cross-detection of EIEC • Cross-sectional design; cannot demonstrate causality • No lactulose: mannitol tests • No blood samples or measure of enteropathogen antibodies
**Schnee and colleagues, 2018 [19]**	PROVIDE (Mirpur, Dhaka, Bangladesh), 2011–2013	Longitudinal community birth cohort	• 521 CU2 • 1,791 MSD stool samples	• GEMS TaqMan (Liu and colleagues, 2016 [8]) enteropathogen surveillance • Serum collected for high sensitivity CRP testing (6, 18, 40, and 53 weeks)	CRP	• *Shigella*/EIEC-attributable MSD was significantly associated with increased CRP levels • CRP increase per attributable episode: 0.24, 95% CI: (0.03, 0.49) • Change in 12-month LAZ per log increase in mean CRP, −0.061, 95% CI: (−0.147, 0.024)	• Not a *Shigella*-specific molecular assay; allows cross-detection of EIEC • Findings may not be generalizable to other LMICs—Bangladesh has a high prevalence of *Shigella* infections in CU2 • Non-diarrheal stool not collected; potential underestimate of relationship of asymptomatic infections and EED markers
**Bona and colleagues, 2019 [29]**	Six cities^b^ from Northeastern Brazil, 2009–2012	Community cross-sectional case-control	1,200 children aged 2–36 mo from low SES communities (presenting in healthcare units or during active surveillance)	• Collected SES, nutritional status, and clinical information • Stool samples tested for *Shigella*/EIEC using PCR • Tested positive samples for 28 VRGs by multiplex PCR • Intestinal inflammation assessed by fecal MPO measures	MPO	Association between carriage of *Shigella* VRGs *ipgB1*, *ipgB2*, *ospF*, *sen*, *virA*, and *virB* and higher levels of MPO	• Small sample size relative to other studies: 60 children were evaluated; 42 were cases • Because of the molecular assays used (Luminex bead-based assays/conventional multiplex PCR), sensitivity was an order of magnitude lower than qPCR
**Simuyandi and colleagues, 2019** **[37]**	Lusaka, Zambia, 2011–2013	Cross-sectional, secondary study	• 234 stool samples collected from children with MSD during rotavirus vaccine administration • Clinical data	• Fecal EE biomarkers • ELISA • Used the qualitative, multiplex PCR-based Luminex x-TAG (Luminex Corporation, Austin, Texas, USA) gastrointestinal pathogen panel to detect 15 enteric pathogens	EE score (weighted sum CALP, MPO, and AAT)	• *Salmonella*, *Shigella*, and *E*. *coli*: largest contributions to the EE score • *Shigella* and *Salmonella* independently associated with increased mean EE score • *Shigella* associated with 0.92 unit increase in EE score (highest pathogen-associated score increase measured in the study) • *Shigella* and EHEC-O157:H7 strongly associated with increase in MPO geometric mean	• Cross-sectional design; cannot demonstrate causality • No blood samples assessed—could provide additional immunological information • No water sanitation and hygiene exposure information collected; could not adjust analysis for these factors

^a^MAL-ED sites: Dhaka, Bangladesh; Fortaleza, Brazil; Vellore, India; Bhaktapur, Nepal; Loreto, Peru; Naushahro Feroze, Pakistan; Venda, South Africa; and Haydom, Tanzania.

^*b*^Cajazeiras (Paraiba), Crato (Ceará), Ouricuri (Pernambuco), Patos (Paraiba), Picos (Piauí), and Sousa (Paraiba).

AAT, α-1-antitrypsin; ACP, α-1-acid glycoprotein; CALP, Calprotectin; CRP, C-reactive protein; CU2, children under age 2 years; CU5, children under age 5 years; CRP, serum C-reactive protein; EE, environmental enteropathy; EED, environmental enteric dysfunction; EHEC-O157:H7, enterohemorrhagic *E*. *coli* O157:H7; EIEC, enteroinvasive *E*. *coli*; ELISA, enzyme-linked immunosorbent assay; GEMS, Global Enteric Multicenter Study; LAZ/HAZ, length/height-for-age Z score; LMIC, low- to middle-income countries; LMZ, lactulose: mannitol excretion ratio Z scores; mo, month(s); MAL-ED, Malnutrition and Enteric Disease Study; MPO, myeloperoxidase; NEO, neopterin; PCR, polymerase chain reaction; qPCR, quantitative PCR; SES, socioeconomic status; VRG, virulence-related gene; WAZ, weight-for-age Z score; WHZ, weight-for-height Z score.

### Animal models of EED and *Shigella*

The specific mechanisms that link enteric infections to linear and ponderal growth faltering remain an area of continuing investigation. A part of this research includes animal models created to evaluate how *Shigella* infections and EED may lead to growth deficits in children. A recent review by Salameh and colleagues (2019) delves into these models extensively [42]. Here, we include 2 such models to show some promising results in this area.

A recent study by Medeiros and colleagues (2019) outlines an animal model designed to explore zinc deficiency effects on mice orally inoculated with *Shigella flexneri* serotype 2, the most common *Shigella* serotype in low-resource settings. The *Shigella*-infected mice exhibited growth impairment, metabolic disturbances, diarrhea, and intestinal inflammation similar to children with shigellosis [43]. A model now exists that can shed light on the mechanisms by which *Shigella* infections may lead to growth impairment. In a mouse model study by Brown and colleagues (2015), malnourished mice exhibited moderate stunting and increased intestinal permeability compared with nourished mice (those on an isocaloric control diet). The malnourished mice gained 30% less weight and had considerably shorter tails, evidence of growth faltering in relation to nutrition levels. Malnourished mice also exhibited decreased growth factor expression and increased expression of intestinal permeability markers, potential targets to explore underlying EED mechanisms further. Malnourished mice were also more susceptible to experimental *Salmonella enterica subsp typhimurium* infection and had higher bacterial burdens than nourished mice [44]. These models could elucidate the exact mechanisms connecting infection by *Shigella* and other enteric pathogens to childhood stunting.

A recent EED and microbiota study in Bangladeshi children found a relationship between specific duodenal bacterial taxa and the degree of stunting exhibited by children [45]. The authors then transferred samples from these children’s microbiome into a mouse model and found support for causal relationships between microbiota assembly within the intestinal tract and EED pathology. However, this study did not report a connection between pathogenic enterobacteria (such as *Shigella*) and stunting. A summary of key conclusions from these studies on EED biomarkers and on the relationship between *Shigella* and EED can be found in Box 4.

**Box 4. Key conclusions**

- ***Shigella* infection has been associated with multiple EED biomarkers, including MPO, AGP, and CRP.**
- ***Shigella* infection has been repeatedly associated with increased MPO expression or detection.**
- **MPO has been associated with decreased linear growth in CU2 and decreased WFA and BMI at age 5.**
- **Animal models can replicate symptoms associated with EED in children and show initial promise to uncover the pathway that links EED to growth faltering.**

### Important considerations regarding the *Shigella*-stunting relationship

#### Cross-detection by molecular assays

Molecular assays amplify a highly conserved region among different species of a particular pathogen to detect multiple versions of a pathogen of interest. Because of this, the assay may amplify other closely related non-target species that contain nearly identical genes. As previously mentioned, many molecular detection assays for *Shigella* will detect bacteria with similar genomes, primarily enteroinvasive *E coli* (EIEC) and, when analyzing 16S ribosomal RNA, *E*. *coli* species. For example, Liu and colleagues (2016) reported that more than 70% of *Shigella*/EIEC infections were due to either *Shigella flexneri* or *Shigella sonnei*, without specifying the identity of the remaining 30% [8]. While *Shigella* and EIEC are traditionally classified as different bacteria, some scientists argue that *Shigella* and EIEC should be classified as the same species due to their indistinguishable genomes (Bona and colleagues, 2019).

According to a recent review of the application of molecular *Shigella* diagnostics in young children from LMICs, the use of these methods has resulted in an approximately 150% increase in *Shigella* burden estimates [33]. The authors indicate that, while some of these molecular tools cannot differentiate between *Shigella* and EIEC, evidence suggests that EIEC is relatively rare, with an upper bound of 8% of these infections being attributable to EIEC rather than *Shigella*. Taking this into consideration, the multiple findings suggesting *Shigella* to be a leading cause of diarrhea remains intact, even in studies where *ipaH* gene copies were detected to characterize *Shigella* burden. In addition, there are other approaches (detection of O-antigen or O-antigen-related genes) to help pinpoint whether the infection is attributable to *Shigella* rather than EIEC. Including these types of assays can improve the accuracy of the molecular assessment of *Shigella* burden, maintaining the use and improving the precision of molecular diagnostics in this realm (see Platts-Mills and Rogawski (2021) [33] and Hazen and colleagues (2016) [46] for a more thorough discussion of this topic).

#### *Shigella* and coinfections

When surveying enteric pathogens in children from community settings, *Shigella* spp are usually detected along with other pathogens. Rogawski and colleagues (2020) explored *Shigella* epidemiology in the MAL-ED cohort, finding that around half of the diarrheal cases attributed to *Shigella* had a potential second diarrheal etiology—ETEC for 53% of cases and *Giardia* for 45.2% of cases [13]. Interestingly, some clinical studies of children from LMICs have found positive associations between certain enteric pathogen pairs. For example, Andersson and colleagues (2018) found a positive association between *Shigella* and either typical or atypical EPEC in CU5 patients with diarrhea from Rwanda and Zambia [47]. The question remains as to whether these co-occurring pathogens can act synergistically to reduce childhood growth. It is also important to consider that these coinfections can complicate future determinations of the magnitude of *Shigella* vaccine impacts on stunting.

#### Dysbiosis and stunting

Dysbiosis is a disruption to the microbiome that results in imbalanced microbiota. Some studies show a link between various types of dysbiosis and stunting rather than because of the presence of specific enteric pathogens. For example, in 2 separate studies, over 80% of children exhibiting stunted growth had SIBO of bacteria usually found in the oropharyngeal cavity [15,21]. Both studies also reported a high prevalence of *Shigella*, but Collard and colleagues (2022) found no difference in its presence between control children and those with stunted growth [15]. Chen and colleagues (2020) found a relationship between specific duodenal bacterial taxa and the degree of stunting exhibited by children, but this relationship did not extend to *Shigella* or other pathogenic bacteria [45]. These findings do not preclude the importance of pathogenic bacteria in LGF, but rather suggest that there is a complex interplay between enteric pathogens, EED, and the gut microbiome that needs to be better characterized.

### Future studies should…

- **Employ molecular assays more specific to *Shigella* or employ additional diagnostic tools to confirm that results are specific to *Shigella*.** This refinement can more accurately assign *Shigella* etiology to diarrheal episodes and will improve understanding of enteric coinfections.
- **Identify diarrheal etiology using standardized diagnostics more often and in different settings and geographic locations to improve child anthropometric surveillance and *Shigella* surveillance.** These actions will improve global and regional *Shigella* burden modeling efforts and better capture socioeconomic, setting (urban/rural)-related, and geographic inequities.
- **Use a variety of study designs, such as longitudinal and human challenge studies, designed to detect true asymptomatic infections and to better understand their associations with growth faltering and other malnutritional outcomes.**
- **Continue exploring the relationship between *Shigella* infections and EED markers and focus on uncovering the underlying mechanism of this relationship.** Improving and standardizing markers will enable better comparisons across different contexts.
- **Apply longitudinal methods to determine *Shigella*’s contribution to non-communicable disease (NCD) risk.** While some studies report an association between diarrhea experienced during infancy and early childhood and a higher childhood NCD risk [43–45], more evidence supports the relationship between childhood malnutrition and adult NCD risk [1–3]. To our knowledge, no published longitudinal studies have quantified the direct association between *Shigella*-attributable diarrhea or infections in childhood with NCDs later in life. This research avenue can shed even more light on the long-term impacts of *Shigella* burden.

**Key Learning Points**

- *Shigella* often has a stronger relationship to linear growth faltering than other enteropathogens; furthermore, higher *Shigella* loads result in greater growth deficits.
- Asymptomatic *Shigella* infections can result in linear growth decrements.
- While *Shigella* infection is less common in infants (0 to 11 months), it can result in severe illness and may be more consequential for future linear growth faltering in infants than for toddlers and children (24 to 59 months).
- *Shigella* infection has been associated with multiple EED biomarkers, including MPO, AGP, and CRP; MPO has been associated with decreased linear growth in CU2 and decreased WFA and BMI at age 5.

**Five Key Papers**

- Rogawski ET, Liu J, Platts-Mills JA, Kabir F, Lertsethtakarn P, Siguas M, et al. Use of quantitative molecular diagnostic methods to investigate the effect of enteropathogen infections on linear growth in children in low-resource settings: longitudinal analysis of results from the MAL-ED cohort study. Lancet Glob Health. 2018;6: e1319–e1328. doi:10.1016/S2214-109X(18)30351-6
- Vonaesch P, Morien E, Andrianonimiadana L, Sanke H, Mbecko J-R, Huus KE, et al. Stunted childhood growth is associated with decompartmentalization of the gastrointestinal tract and overgrowth of oropharyngeal taxa. Proc Natl Acad Sci U S A. 2018;115: E8489–E8498. doi:10.1073/pnas.1806573115
- Rogawski McQuade ET, Shaheen F, Kabir F, Rizvi A, Platts-Mills JA, Aziz F, et al. Epidemiology of Shigella infections and diarrhea in the first two years of life using culture-independent diagnostics in 8 low-resource settings. Talaat K, editor. PLoS Negl Trop Dis. 2020;14: e0008536. doi:10.1371/journal.pntd.0008536
- Kotloff KL, Nasrin D, Blackwelder WC, Wu Y, Farag T, Panchalingham S, et al. The incidence, aetiology, and adverse clinical consequences of less severe diarrhoeal episodes among infants and children residing in low-income and middle-income countries: a 12-month case-control study as a follow-on to the Global Enteric Multicenter Study (GEMS). Lancet Glob Health. 2019;7: e568–e584. doi:10.1016/S2214-109X(19)30076-2
- Nasrin D, Blackwelder WC, Sommerfelt H, Wu Y, Farag TH, Panchalingam S, et al. Pathogens associated with linear growth faltering in children with diarrhea and impact of antibiotic treatment: The Global Enteric Multicenter Study. J Infect Dis. 2021 [cited 6 Oct 2021]. doi:10.1093/infdis

## Supporting information

S1 Table Multisite studies showing relationships between diarrhea of multiple etiologies and child growth faltering or environmental enteric dysfunction (EED). (DOCX)
