# Supplementary material for: Shigella and childhood stunting: Evidence, gaps, and future research directions
Source: PLoS Negl Trop Dis. 2023 Sep 12;17(9):e0011475. doi: 10.1371/journal.pntd.0011475 (PMC10497124; doi:10.1371/journal.pntd.0011475)
Supplement: S1 Table — (DOCX) [file pntd.0011475.s001.docx]

**S1 Table. Multisite studies showing relationships between diarrhea of multiple etiologies and child growth faltering or EED**

| Author, Year | Study Site(s), Period | Study Design | Study Population | Methods | Growth Faltering Related Measure | Main Outcomes Pertinent to Review | Limitations |
| --- | --- | --- | --- | --- | --- | --- | --- |
| *MAL-ED* |  |  |  |  |  |  |  |
| Rogawski McQuade et al., (2020)[1] | MAL-ED sites,* 2009–2012 | Secondary stool analysis from longitudinal community birth cohort study | 41,450 stool samples from 1,715 CU2 | - Monthly stool and diarrheal stool samples - GEMS TaqMan (ThermoFisher, Waltham, MA, USA) [2,3] for enteropathogen surveillance | Monthly WAZ and LAZ | - Diarrheal stool samples had significantly higher *Shigella* prevalence than asymptomatic stool samples - Prevalence and *Shigella* quantity increased with age - *Shigella* presented differently according to age | - Not a *Shigella*-specific molecular assay; allows cross-detection of EIEC - Not all diarrhea episodes tested; potentially underestimated *Shigella* incidence - Most stools coinfected with multiple enteropathogens; difficult to determine if *Shigella* was the primary or secondary cause of diarrhea - *Shigella* burden variation among sites makes it difficult to generalize findings to other LMIC sites |
| MAL-ED investigators, (2017A) [4] | MAL-ED sites*, 2009–2014 | Longitudinal community birth cohort | - 1,197 of 1,868 CU2 - 23,629 monthly observations | - Methods from MAL-ED Investigators 2014 [5] - Monthly length and weight, sociodemographic factors, maternal illness reports, use of antibiotics; child feeding practices; dietary intake starting at 9 mos. - Samples collected: blood (7,15, 24 mo.) and urine (3,6,9, 15 mo.); monthly stool samples in the first y, quarterly thereafter. - Pathogen detection methods: bacteriological culture/ELISA for non-*E. coli* bacteria [6] | - Monthly WAZ and LAZ - MPO, NEO, AAT, and LMZ scores - ACP | - Children at all sites (except Brazil) had linear growth faltering - Neonatal and maternal factors (enrollment weight, maternal height) early determinants of lower LFA and until age 2 - By age 2, the average number of enteropathogens in asymptomatic stool, socioeconomic status, and dietary intake were important contributors to lower LFA; became increasingly important as child age increased - Gut inflammation biomarkers (MPO, NEO) not associated with lower LFA - AAT and ACP associated with lower LFA in younger children, relationship changed as children aged - Children in lower LAZ category had a higher LMZ score, but not statistically significant | - Complex covariance between risk factors - Pakistan excluded from analyses (bias in LFA measurements) - Missing data: lost to follow-up and dropouts (esp. from Brazil) 69% of the original cohort had longitudinal data - Some factors averaged across age—may not reflect true temporal associations - Limited generalizability: some of the populations may have been better off than others - Gestational age at delivery was not assessed and may affect LAZ assessment |
| MAL-ED investigators, (2017B) [7] | MAL-ED sites* (excluding Nepal), 2009–2014 | Longitudinal community birth cohort | - 1,197 of 1,868 CU2 - 23,629 monthly observations | - Sampling and detection methods same as MAL-ED Investigators 2014 [5] - Linear piecewise spline model to quantify associations of factors with growth velocity | - Monthly WAZ and LAZ | - Diarrhea did not have a statistically significant effect on growth - On average, children with higher enteropathogen exposure were shorter 1.21±0.33 cm (P < 0.001; 0.39 [LAZ]) exposures and lighter 0.08±0.15 kg (p=0.60; 0.08 [WAZ]) at 2 y than children with lower exposure - Detected subclinical *Campylobacter* and EAEC infections, and they were associated with growth deficits by 24 mo | - Unable to ascertain causality given study design - Limited sample size due to Pakistan removal and incomplete/missing data (from Brazil) - Unable to assess all enteropathogens or differentiate persistent from new infections to compare child growth impacts - Not able to assess breast milk intake or diet on child growth impacts |
| Kosek & MAL-ED investigators (2017) [8] | MAL-ED sites,* 2009–2014 | Longitudinal community birth cohort | - 1,253 CU2 - 22,846 non-diarrheal stool samples | - Sampling and detection methods same as MAL-ED Investigators 2014 [5] - Direct acyclic graph - CODA (COmmunity DiarrheA) diarrheal severity score - Classified enteropathogens into five groups based on pathophysiology | - Monthly WAZ and LAZ - MPO, NEO, AAT, and LMZ scores - ACP | - Asymptomatic stool samples containing enteropathogens were associated with higher fecal MPO levels - Stronger signals of inflammation in fecal biomarkers were associated with *Shigella*, *Yersinia*, and *Campylobacter* spp - Trend for EED biomarker concentrations to decrease with increasing age - None of the EED biomarkers had large mean effects on the average LAZ or WAZ | - Only a subset of MAL-ED children with complete data were included in the direct acyclic graph model - Small effect sizes of biomarkers to growth - Growth faltering attributed to EED biomarkers small compared with the accumulated growth deficits in the study population |
| Platts-Mills et al., (2015) [9] | MAL-ED sites,* 2007–2009 | Longitudinal community birth cohort | - 7,318 diarrheal and 24,310 non-diarrheal stools - 2,145 CU2 - 81% of participants have 2 y of follow-up data | - Sampling and detection methods same as MAL-ED Investigators 2014 [5] - Calculated diarrheal severity score (incorporates vomiting, fever, frequency, and dehydration) | - N/A | - *Shigella* was one of the pathogens with highest burden pathogen in y 2 across sites - *Shigella* was associated with dysentery, dehydration, hospitalization, fever during y 2 - *Rotavirus* and *Shigella* ranked the highest in severity of symptoms (rotavirus [OR: 2.30 per unit increase severity score]; *Shigella* [OR: 1.48 per unit increase severity score]) - *Shigella* associated with persistent diarrhea in y 1 and y 2 - *Shigella* associated with severe dysentery and long diarrhea duration - Number and diversity of pathogens associated with community diarrhea was high | - Not powered to identify all associations between pathogens and diarrhea at individual sites - Potential bias in burden estimates - Difficult to measure short episodes/durations of diarrhea - Use of a modified severity score may affect generalizability to other LMICs - Culture methods may underestimate bacterial presence |
| *GEMS* |  |  |  |  |  |  |  |
| Kotloff et al., (2019) [10] | GEMS sites^b^ (excluding Kenya), 2011–2012 | 1-y prospective, age-stratified, matched case-control study  follow-on to GEMS | - 2,368 CU5 with MSD - 3,174 CU5 with LSD, and one to three randomly selected community control children without diarrhea matched to cases with MSD (n=3,597) or LSD (n=4,236) | - Collected clinical and epidemiological data, anthropometric measurements, and fecal samples at enrollment and 60-day follow-up - CU5 who sought care at a sentinel hospital or health center during a 12-mo period were screened for diarrhea | - HAZ | - *Shigella*-attributable diarrhea increased with age; significantly associated with MSD or LSD, or both, at two sites during infancy (Bangladesh and Pakistan) and five sites (all sites except Mali) in each of the older strata - *Shigella* spp: third highest incidence for MSD among infants (1.3 episodes per 100 child y; 0.3–2.3), second: toddlers (2.4 episodes per 100 child y; 0.6–4.1), first: older children (0–7 episodes per 100 child y, 0.1–1.3) | - 1 year enrollment, limiting statistical power for comparisons - LSD cases did not have unique clinical parameters - Incident cases included all episodes of diarrhea with a wide range of severity - The study estimates of the overall incidence of acute, new-onset diarrheal disease (i.e., MSD plus LSD) were considerably lower, may be underestimated - Potential reporting bias/underreporting of milder diarrheal episodes during the previous week |
| Brander et al., 2019 [11] | GEMS sites**, 2007–2009 | Post hoc analysis of CU2 MSD cases in GEMS cohort | 6,203 CU2 children with MSD | - Log-binomial regression analysis of GEMS data to understand clinical SES factors associated with stunting (loss of ≥ 0.5 LAZ) | - LAZ | - Young age, acute malnutrition, severe MSD, and SES factors were related to acute linear growth loss/stunting following MSD - Infants (< 1y) with MSD experienced more significant linear growth deficits than children aged 12 to 23 mo - Children aged 6 to 12 mo experienced a greater LAZ loss (0.07 LAZ) than children aged 12 to 23 mo at follow-up - Children aged 0 to 6 mo with MSD exhibited more severe linear growth faltering than children 12 to 23 mo at follow-up | - Data on birth size, HIV status, and before/after diarrhea episodes were not assessed/available - Analysis only evaluated acute/short-term effects (2–3 mo); no assessment of long-term effects |
| Berendes et al., (2018) [12] | Kenya GEMS site, 2008–2012 | Matched case-control study | - Cases: CU5 with MSD - Controls: CU5 w/o reported diarrhea 7 days before visit | - Collected clinical and epidemiological data, anthropometric measurements, and fecal samples at enrollment and 60-day follow-up, - Used a caretaker memory aid for recording diarrheal incidence - Stool specimen analyzed by microbial methods; focused on MSD pathogens (rotavirus, *Cryptosporidium*, *Shigella* spp, typical EPEC, heat stable ETEC, non-typhoidal *Salmonella* spp) | - HAZ | - 19% of controls had one enteric pathogen associated with MSD at enrollment - After enrollment, 27% reported diarrhea in 7 days (39% in 14 days) - Asymptomatic and symptomatic controls had similar carriage of MSD pathogens at enrollment - Controls with MSD bacteria had significantly higher odds of stunting at follow-up, regardless of showing symptoms, as compared with controls without MSD bacteria | - Data on MSD symptoms for controls were not collected - Results from controls are not generalizable to other LMICs - No symptom onset/timing/duration data collected, limiting conclusions about etiologic cause - Potential recall bias |
| Lindsay et al., (2015) [13] | 4 GEMS sites (The Gambia, Mali, Bangladesh, and Kenya), 2007–2009 | Secondary analysis of stool samples and risk factors | - 2,611 fecal samples from CU5; (matched sets 1,181) - Around half of the children were male and from Kenya (56% male, 50% from Kenya) | - MSD - Molecular assay: SYBR green qPCR assay. - Samples over 14,000 *ipaH* copies considered “high shedding” | - HAZ | - *Shigella* isolated most often in 6 to 11 mo or 12 to 23 mo age groups - *Shigella* quantity did not differ between different nutritional statuses - Age and site: statistically significant effect modifiers of *Shigella* quantity and MSD - The highest AF: in 12–23 mo for The Gambia and Mali and 24–59 mo for Kenya and Bangladesh - MSD and high *Shigella* load peaked at 24–35 mo | - Convenience sample: did not age stratify - Self-reports may have cultural and reporting biases that make estimates unreliable - Multiplex approach may have been able to establish specific etiology and estimate AFs - Not designed to evaluate the differences between cases and controls with *ipaH* values ≥ 14,000 - Not powered to detect differences between these groups - Differences by site could be due to some unmeasured confounders (e.g., HIV status) |
| Kotloff et al., (2013) [14] | GEMS sites^b^, 2007–2009 | Prospective, age-stratified, matched case-control study | Cases: 9,439 CU5 with MSD Controls: 13,129 CU5 | - Collected clinical and epidemiological data, anthropometric measurements, and fecal samples at enrollment and 60-day follow-up - Microbiological methods to detect *Shigella* (bacterial culture) | - HAZ | - Four pathogens responsible for most pathogen-attributable MSD cases: rotavirus, *Cryptosporidium*, *Shigella, and* heat-stable toxin producing ETEC - Excluding Bangladesh, *Shigella*-attributable fraction; 2%–7.6% in infants, 2%–12.8% in toddlers, and 2%–14.9% in children 24–59 mo - In Bangladesh, *Shigella*-attributable fraction = 67.6 in children aged 24–59 mo | - Enteropathogens are commonly detected in asymptomatic controls, which underestimates AF - Coinfections and comorbidities not considered—may have contributed to MSD and adverse outcomes - MSD incidence may be limited due to low hospital use by children in the study - Clinical criteria for MSD varied across sites |

*AAT: α-1-antitrypsin; ACP: α-1-acid glycoprotein; CU2: children under age 2 years; CU5: children under age 5 years; EED: Environmental enteric dysfunction; EAEC: enteroaggregative E. coli; EIEC: enteroinvasive E. coli; ELISA: Enzyme-linked immunosorbent assay;* *EPEC: enteropathogenic E. coli; ETEC: enterotoxigenic Escherichia coli; GEMS: Global Enteric Multicenter Study; LAZ/HAZ: length/height-for-age Z score; LSD: less severe diarrhea; LMIC: Low to middle income countries; LMZ: lactulose: mannitol excretion ratio Z scores; mo: month(s); MAL-ED: Malnutrition and Enteric Disease Study; MPO: myeloperoxidase; MSD: moderate-to-severe diarrhea; NEO: neopterin; OR: odds ratio; qPCR: quantitative polymerase chain reaction; SES: socioeconomic status; WAZ: weight-for-age Z score*

^a^*MAL-ED sites: Dhaka, Bangladesh; Fortaleza, Brazil; Vellore, India; Bhaktapur, Nepal; Loreto, Peru; Naushahro Feroze, Pakistan; Venda, South Africa; and Haydom, Tanzania.*

***^b^****GEMS sites: Bamako, Mali; Manhiça, Mozambique; Nyanza Province, Kenya; Basse Santa Su, The Gambia; Mirzapur, Bangladesh; Kolkata, India; and Bin Qasim Town, Karachi, Pakistan.*

# **References**

1. Rogawski McQuade ET, Shaheen F, Kabir F, Rizvi A, Platts-Mills JA, Aziz F, et al. Epidemiology of Shigella infections and diarrhea in the first two years of life using culture-independent diagnostics in 8 low-resource settings. Talaat K, editor. PLoS Negl Trop Dis. 2020;14: e0008536. doi:10.1371/journal.pntd.0008536

2. Liu J, Gratz J, Amour C, Kibiki G, Becker S, Janaki L, et al. A Laboratory-Developed TaqMan Array Card for Simultaneous Detection of 19 Enteropathogens. J Clin Microbiol. 2013;51: 472–480. doi:10.1128/JCM.02658-12

3. Liu J, Platts-Mills JA, Juma J, Kabir F, Nkeze J, Okoi C, et al. Use of quantitative molecular diagnostic methods to identify causes of diarrhoea in children: a reanalysis of the GEMS case-control study. The Lancet. 2016;388: 1291–1301. doi:10.1016/S0140-6736(16)31529-X

4. MAL-ED Network Investigators. Childhood stunting in relation to the pre- and postnatal environment during the first 2 years of life: The MAL-ED longitudinal birth cohort study. PLOS Med. 2017;14: e1002408. doi:10.1371/journal.pmed.1002408

5. The MAL-ED Network Investigators. The MAL-ED Study: A Multinational and Multidisciplinary Approach to Understand the Relationship Between Enteric Pathogens, Malnutrition, Gut Physiology, Physical Growth, Cognitive Development, and Immune Responses in Infants and Children Up to 2 Years of Age in Resource-Poor Environments. Clin Infect Dis. 2014;59: S193–S206. doi:10.1093/cid/ciu653

6. Houpt E, Gratz J, Kosek M, Zaidi AKM, Qureshi S, Kang G, et al. Microbiologic methods utilized in the MAL-ED cohort study. Clin Infect Dis Off Publ Infect Dis Soc Am. 2014;59 Suppl 4: S225-232. doi:10.1093/cid/ciu413

7. MAL-ED Network Investigators. Relationship between growth and illness, enteropathogens and dietary intakes in the first 2 years of life: findings from the MAL-ED birth cohort study. BMJ Glob Health. 2017;2: e000370. doi:10.1136/bmjgh-2017-000370

8. Kosek MN, MAL-ED Network Investigators. Causal Pathways from Enteropathogens to Environmental Enteropathy: Findings from the MAL-ED Birth Cohort Study. EBioMedicine. 2017;18: 109–117. doi:10.1016/j.ebiom.2017.02.024

9. Platts-Mills JA, Babji S, Bodhidatta L, Gratz J, Haque R, Havt A, et al. Pathogen-specific burdens of community diarrhoea in developing countries: a multisite birth cohort study (MAL-ED). Lancet Glob Health. 2015;3: e564-575. doi:10.1016/S2214-109X(15)00151-5

10. Kotloff KL, Nasrin D, Blackwelder WC, Wu Y, Farag T, Panchalingham S, et al. The incidence, aetiology, and adverse clinical consequences of less severe diarrhoeal episodes among infants and children residing in low-income and middle-income countries: a 12-month case-control study as a follow-on to the Global Enteric Multicenter Study (GEMS). Lancet Glob Health. 2019;7: e568–e584. doi:10.1016/S2214-109X(19)30076-2

11. Brander RL, Pavlinac PB, Walson JL, John-Stewart GC, Weaver MR, Faruque ASG, et al. Determinants of linear growth faltering among children with moderate-to-severe diarrhea in the Global Enteric Multicenter Study. BMC Med. 2019;17: 214. doi:10.1186/s12916-019-1441-3

12. Berendes DM, O’Reilly CE, Kim S, Omore R, Ochieng JB, Ayers T, et al. Diarrhoea, enteric pathogen detection and nutritional indicators among controls in the Global Enteric Multicenter Study, Kenya site: an opportunity to understand reference populations in case-control studies of diarrhoea. Epidemiol Infect. 2018; 1–9. doi:10.1017/S0950268818002972

13. Lindsay B, Saha D, Sanogo D, Das SK, Omore R, Farag TH, et al. Association Between Shigella Infection and Diarrhea Varies Based on Location and Age of Children. Am J Trop Med Hyg. 2015;93: 918–924. doi:10.4269/ajtmh.14-0319

14. Kotloff KL, Nataro JP, Blackwelder WC, Nasrin D, Farag TH, Panchalingam S, et al. Burden and aetiology of diarrhoeal disease in infants and young children in developing countries (the Global Enteric Multicenter Study, GEMS): a prospective, case-control study. The Lancet. 2013;382: 209–222. doi:10.1016/S0140-6736(13)60844-2
